# Supplementary material for: Comparative Evaluation of Solid-phase and Membrane Mimetic Strategies in Membrane Proteome Coverage and Disease-State Analysis
Source: Mol Cell Proteomics. 2025 Dec 19;25(2):101496. doi: 10.1016/j.mcpro.2025.101496 (PMC12858350; doi:10.1016/j.mcpro.2025.101496)
Supplement: Supplemental Data [file mmc1.pdf]

Comparative Evaluation of Solid-Phase and Membrane Mimetic Strategies in Membrane Proteome Coverage and Disease-State Analysis

Frank Antony, Ashim Bhattacharya, Hiroyuki Aoki, Rupinder S. Jandu, Abdualrahman M. Abdualkader, Rami Al Batran, Mohan Babu, Franck Duong van Hoa

**Supplemental Figure 1**	Resolution of mLiver Workflow Membrane Proteome by 15% SDS-PAGE
**Supplemental Figure 2**	Intra-Method Correlation Plots
**Supplemental Figure 3**	Inter-Method Correlation Plots
**Supplemental Figure 4**	Pairwise Wilcoxon Rank-Sum Testing with Benjamini-Hochberg Correction of GRAVY Scores
**Supplemental Figure 5**	Distribution of IMPs Across Quartiles of Protein Abundance
**Supplemental Figure 6**	Distribution of Detected Proteins by Molecular Mass and Comparison of Relative Protein Enrichment Between the FASP and S-Trap Workflows.
**Supplemental Figure 7**	Relative Proportion of pIMPs by Molecular Weight Across the Membrane Mimetic Workflows
**Supplemental Figure 8**	Shared and Unique Detection for SLC Transporters Across Membrane Mimetic Workflow and Transmembrane Segment Distribution

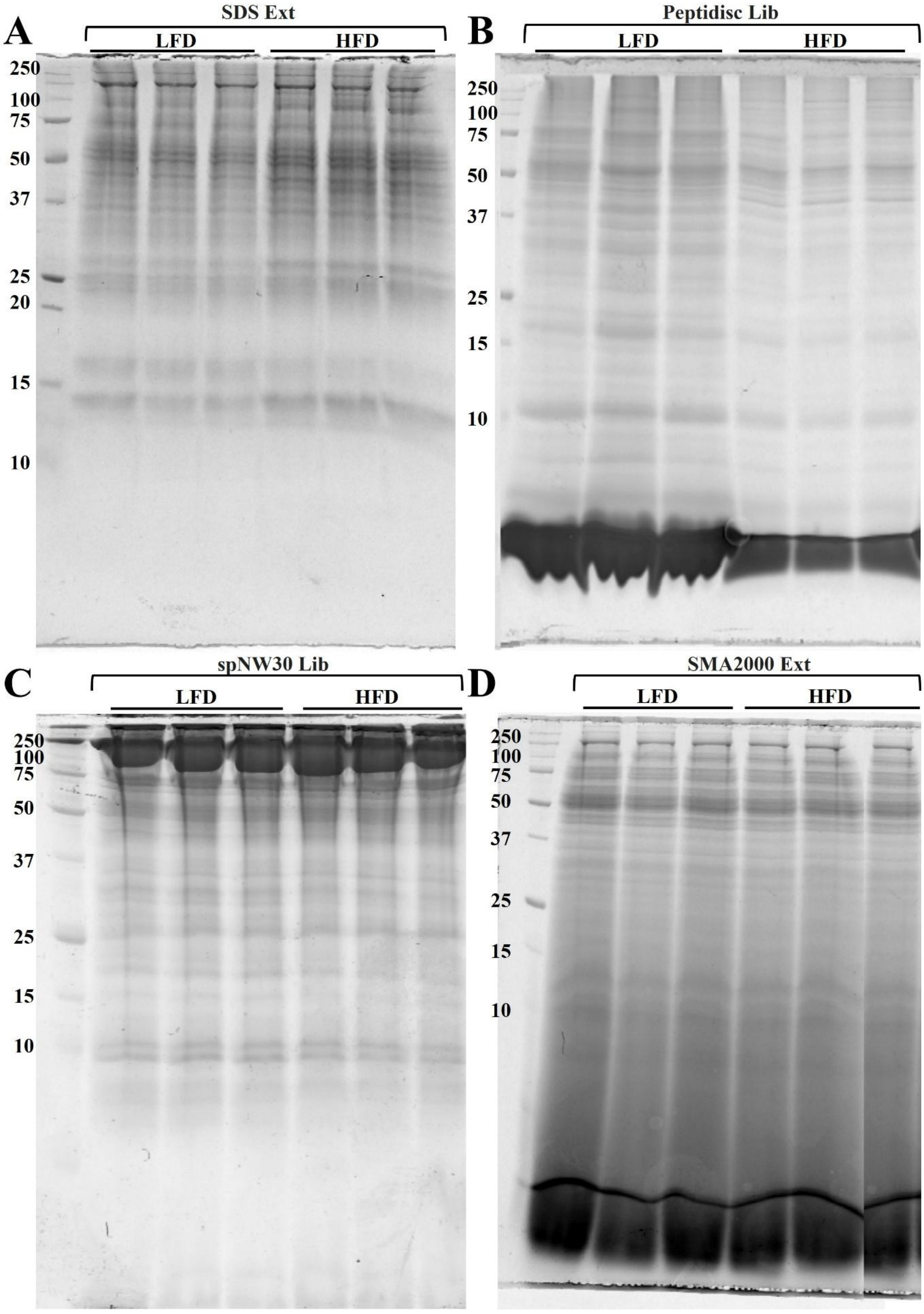
A
SDS Ext
LFD
HFD
250
100
75
50
37
25
20
15
10
B
Peptidisc Lib
LFD
HFD
250
100
75
50
37
25
15
10
C
spNW30 Lib
LFD
HFD
250
100
75
50
37
25
15
10
D
SMA2000 Ext
LFD
HFD
250
100
75
50
37
25
15
10

Supplemental Figure 1. Resolution of mLiver Workflow Membrane Proteome by 15% SDS-PAGE

(A) 15% SDS-PAGE gel showing proteins extracted with 1% SDS from the LFD and HFD mLiver crude membranes, prior to processing with SP3, SP4, FASP, or S-Trap workflows.

(B) Peptidisc library from LFD and HFD mLiver.

(C) spNW30 library from LFD and HFD mLiver.

(D) SMA2000 extracts from LFD and HFD mLiver crude.

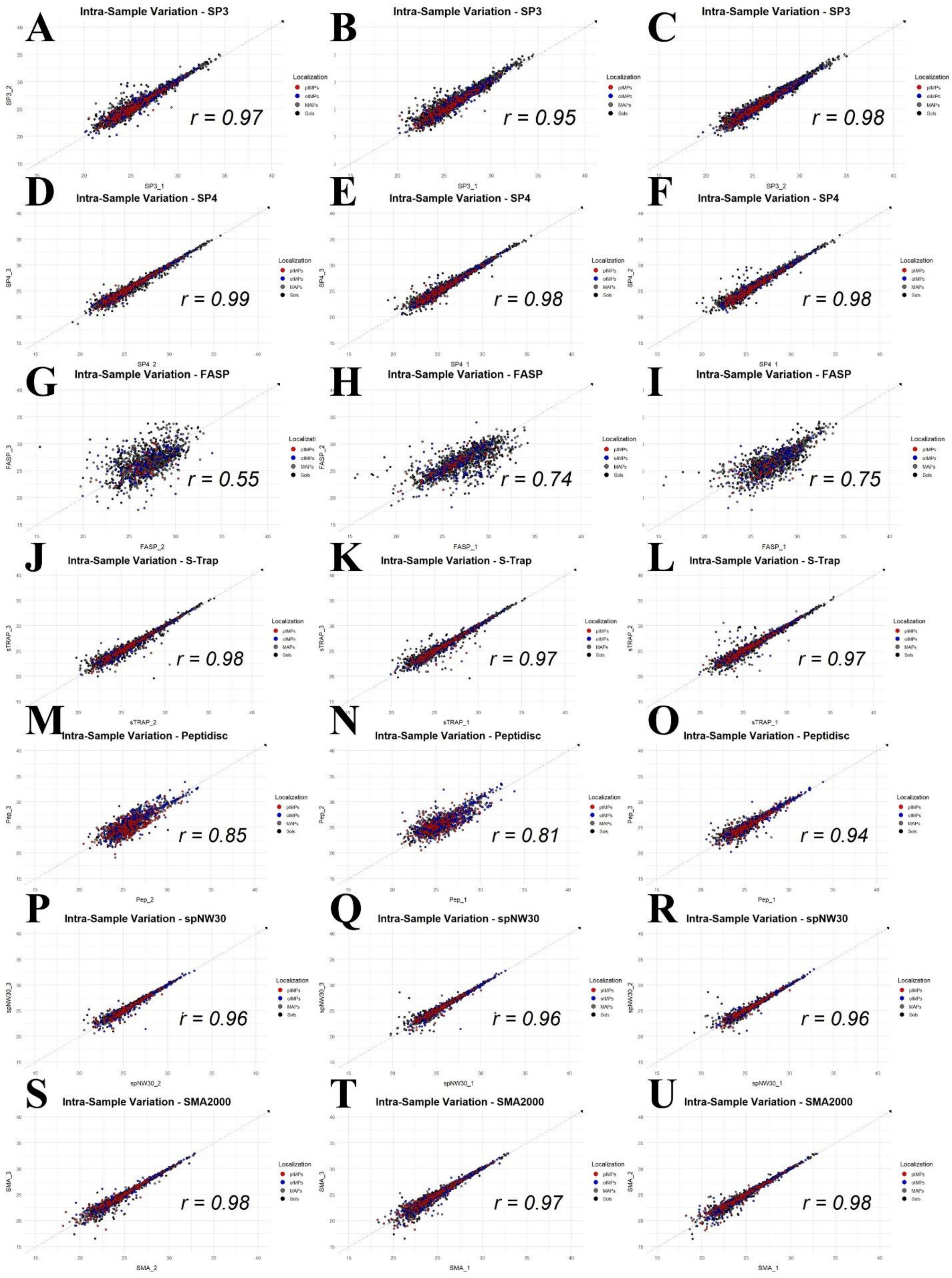
A
Intra-Sample Variation - SP3
r = 0.97
SP3_2
SP3_1
B
Intra-Sample Variation - SP3
r = 0.95
SP3_1
C
Intra-Sample Variation - SP3
r = 0.98
SP3_2
D
Intra-Sample Variation - SP4
r = 0.99
SP4_3
SP4_2
E
Intra-Sample Variation - SP4
r = 0.98
SP4_3
SP4_1
F
Intra-Sample Variation - SP4
r = 0.98
SP4_2
SP4_1
G
Intra-Sample Variation - FASP
r = 0.55
FASP_3
FASP_2
H
Intra-Sample Variation - FASP
r = 0.74
FASP_2
FASP_1
I
Intra-Sample Variation - FASP
r = 0.75
FASP_1
J
Intra-Sample Variation - S-Trap
r = 0.98
sTRAP_3
sTRAP_2
K
Intra-Sample Variation - S-Trap
r = 0.97
sTRAP_3
sTRAP_1
L
Intra-Sample Variation - S-Trap
r = 0.97
sTRAP_2
sTRAP_1
M
Intra-Sample Variation - Peptidisc
r = 0.85
Pep_3
Pep_2
N
Intra-Sample Variation - Peptidisc
r = 0.81
Pep_2
Pep_1
O
Intra-Sample Variation - Peptidisc
r = 0.94
Pep_3
Pep_1
P
Intra-Sample Variation - spNW30
r = 0.96
spNW30_3
spNW30_2
Q
Intra-Sample Variation - spNW30
r = 0.96
spNW30_3
spNW30_1
R
Intra-Sample Variation - spNW30
r = 0.96
spNW30_2
spNW30_1
S
Intra-Sample Variation - SMA2000
r = 0.98
SMA_3
SMA_2
T
Intra-Sample Variation - SMA2000
r = 0.97
SMA_3
SMA_1
U
Intra-Sample Variation - SMA2000
r = 0.98
SMA_2
SMA_1
Localization
pIMPs
oIMPs
MAPs
Sols

Supplemental Figure 2. Intra-Method Correlation Plots

(A-U) Pairwise scatter plots showing MaxLFQ intensity correlations between the three biological replicates for each workflow (A–C: SP3, D–F: SP4, G–I: FASP, J–L: S-Trap, M–O: Peptidisc, P–R: spNW30, and S–U: SMA2000). For each pair of replicates, Pearson correlation coefficients (r-values) were computed using the cor() function in R with method = "pearson" and missing values handled by pairwise deletion (use = "pairwise.complete.obs"). Protein classes are color-coded: pIMPs in red, oIMPs in blue, MAPs in grey, and SPs in black. A dotted diagonal line indicates the 1:1 correlation reference. Plots were generated using the ggplot2 package.

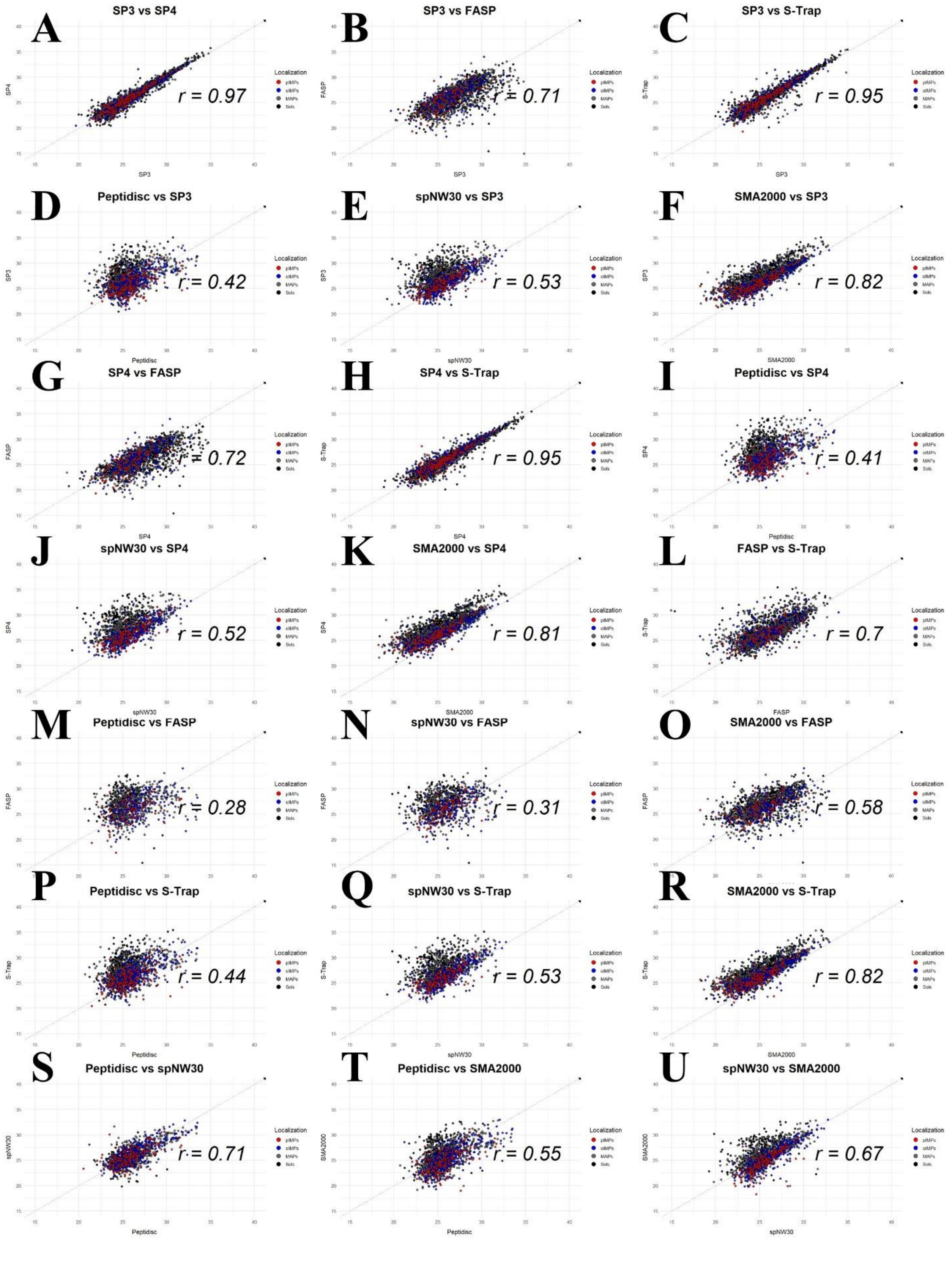
A
SP3 vs SP4
r = 0.97
B
SP3 vs FASP
r = 0.71
C
SP3 vs S-Trap
r = 0.95
D
Peptidisc vs SP3
r = 0.42
E
spNW30 vs SP3
r = 0.53
F
SMA2000 vs SP3
r = 0.82
G
SP4 vs FASP
r = 0.72
H
SP4 vs S-Trap
r = 0.95
I
Peptidisc vs SP4
r = 0.41
J
spNW30 vs SP4
r = 0.52
K
SMA2000 vs SP4
r = 0.81
L
FASP vs S-Trap
r = 0.7
M
Peptidisc vs FASP
r = 0.28
N
spNW30 vs FASP
r = 0.31
O
SMA2000 vs FASP
r = 0.58
P
Peptidisc vs S-Trap
r = 0.44
Q
spNW30 vs S-Trap
r = 0.53
R
SMA2000 vs S-Trap
r = 0.82
S
Peptidisc vs spNW30
r = 0.71
T
Peptidisc vs SMA2000
r = 0.55
U
spNW30 vs SMA2000
r = 0.67
Localization
pIMPs
oIMPs
MAPs
Sols

Supplemental Figure 3. Inter-Method Correlation Plots

(A-U) Pairwise scatter plots comparing MaxLFQ intensities between workflows. The replicate with the highest number of protein identifications in each workflow was selected for analysis. Correlation was calculated across shared proteins between each method pair. Pearson correlation GRAVYcoefficients (r-values) were computed using the cor() function in R with method = "pearson" and missing values handled by pairwise deletion (use = "pairwise.complete.obs"). Points are color-coded by protein class: pIMPs (red), oIMPs (blue), MAPs (grey), and SPs (black). A dotted diagonal line indicates the 1:1 reference. All plots were generated using the ggplot2 package.

Pairwise Wilcoxon Test (BH-adjusted)

	SP3	SP4	FASP	S-Trap	Peptidisc	spNW30	SMA2000
SMA2000	****	****	****	ns	****	****	
spNW30	****	****	****	****	ns		****
Peptidisc	****	****	****	****		ns	****
S-Trap	****	****	****		****	****	ns
FASP	****	****		****	****	****	****
SP4	****		****	****	****	****	****
SP3		****	****	****	****	****	****

ns = not significant
* p < 0.05
** p < 0.01
*** p < 0.001
**** p < 0.0001

Supplemental Figure 4. Pairwise Wilcoxon Rank-Sum Testing with Benjamini-Hochberg Correction of GRAVY Scores

(A) Wilcoxon rank-sum tests were performed between all method pairs with p-values adjusted using the Benjamini–Hochberg (BH) procedure. Significance levels: ns = not significant; * $p < 0.05$; ** $p < 0.01$; *** $p < 0.001$; **** $p < 0.0001$. GRAVY scores were calculated as described in (A). Plot generated using ggplot in R. Statistical analysis performed using the rstatix package in R.

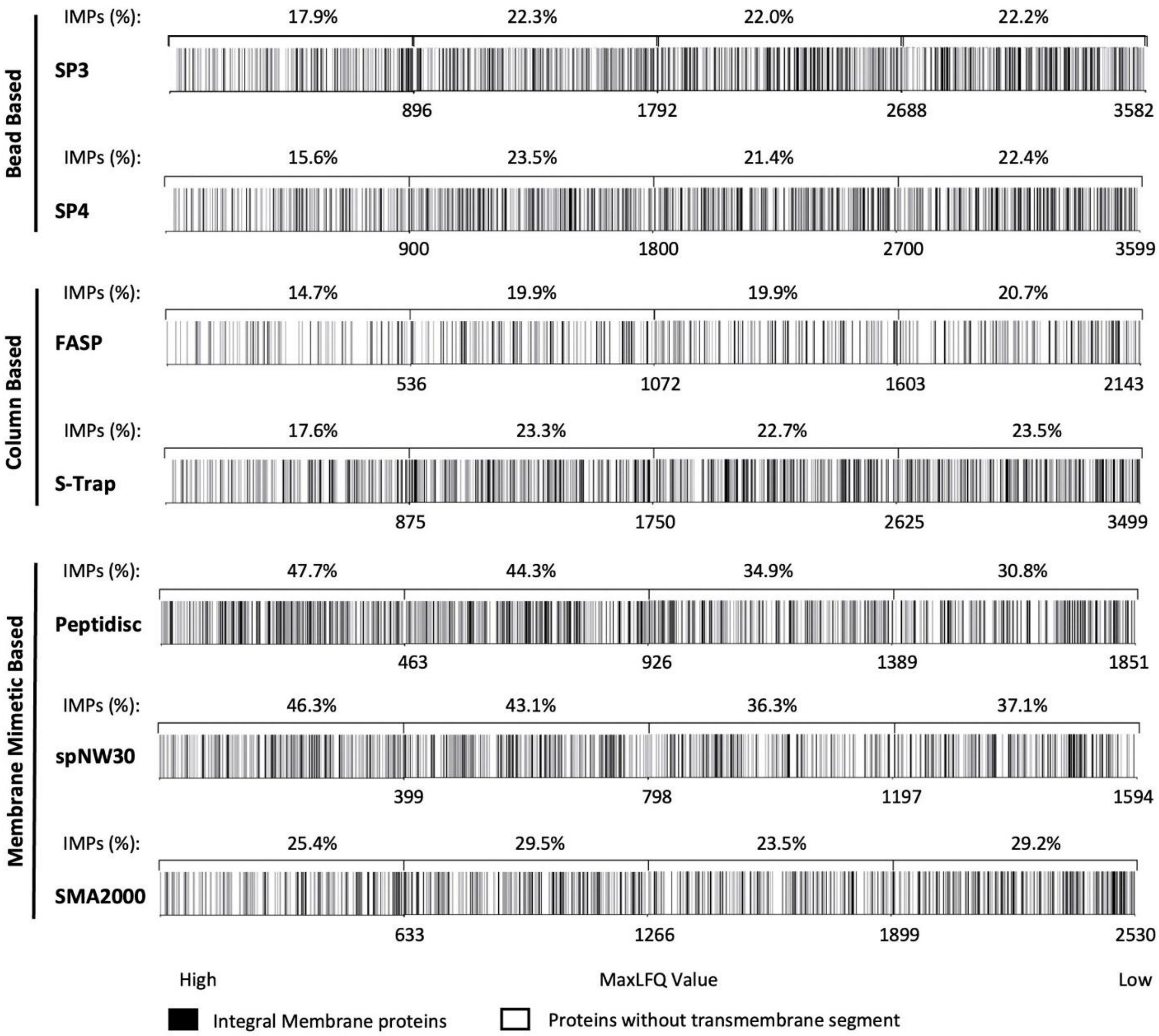

Supplemental Figure 5. Distribution of IMPs Across Quartiles of Protein Abundance

(A-C) Distribution of IMPs across quartiles of protein abundance (Max LFQ Intensity) for (A) SP3 and SP4, (B) FASP and S-Trap, (C) Peptidisc, spNW30, and SMA2000. Quartiles are ordered from highest (left) to lowest (right) intensity. Plotted with GraphPad Prism 10.

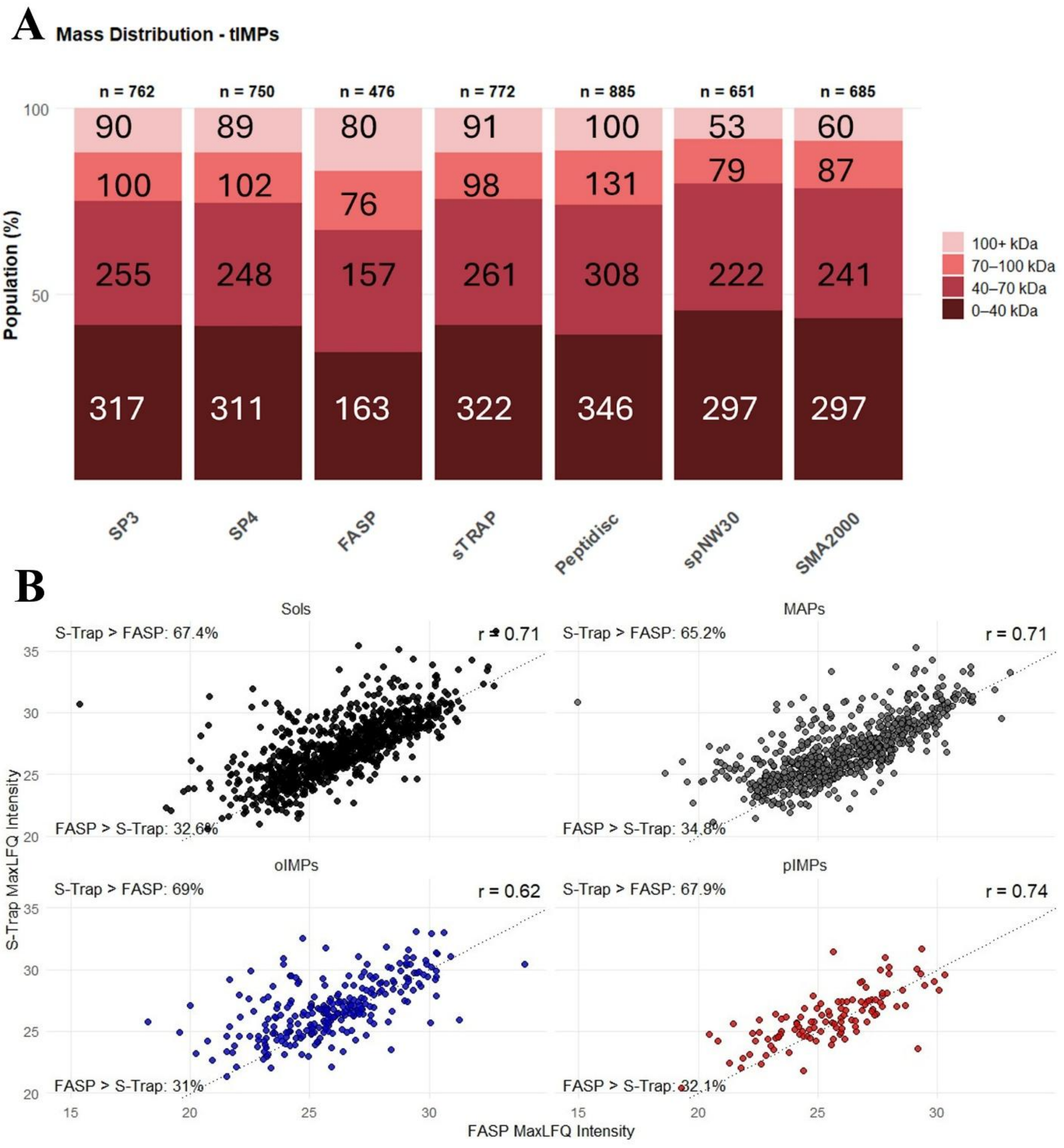

Supplemental Figure 6. Distribution of Detected Proteins by Molecular Mass and Comparison of Relative Protein Enrichment Between the FASP and S-Trap Workflows. **(A)** Stacked bar plot illustrating the relative distribution of proteins captured across four molecular mass bins (0–40 kDa, 40–70 kDa, 70–100 kDa, and >100 kDa) for the seven

proteomic workflows applied to healthy mouse liver samples. Protein masses were obtained from the FragPipe output, and visualization was generated in R using the ggplot2 package.

(B) Pairwise scatter plots showing the relationship between MaxLFQ intensities across workflows and the relative enrichment of proteins captured by the FASP and S-Trap methods in healthy mouse liver samples.

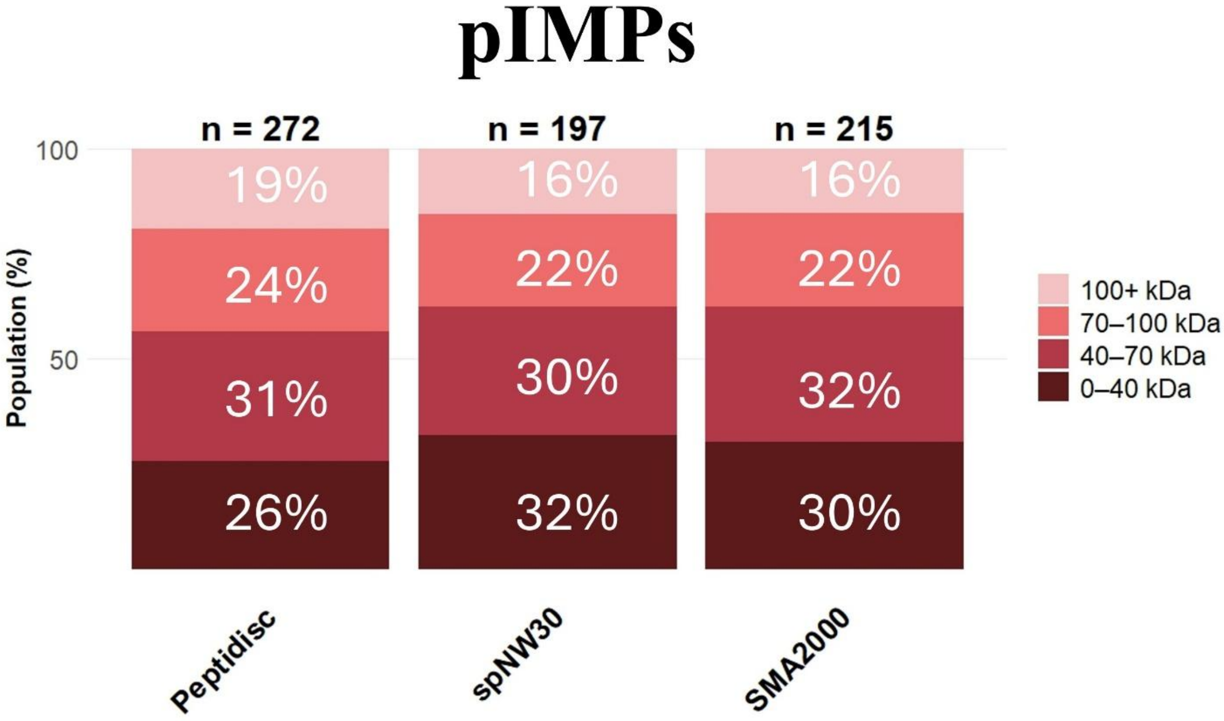

Supplemental Figure 7. Relative Proportion of pIMPs by Molecular Weight Across the Membrane Mimetic Workflows

Stacked barplot showing the percent distribution of pIMPs captured in the three membrane mimetic workflows binned by molecular weight. Plotted using ggplot2 R package. The total number (n) of pIMPs analysis is indicated.

A

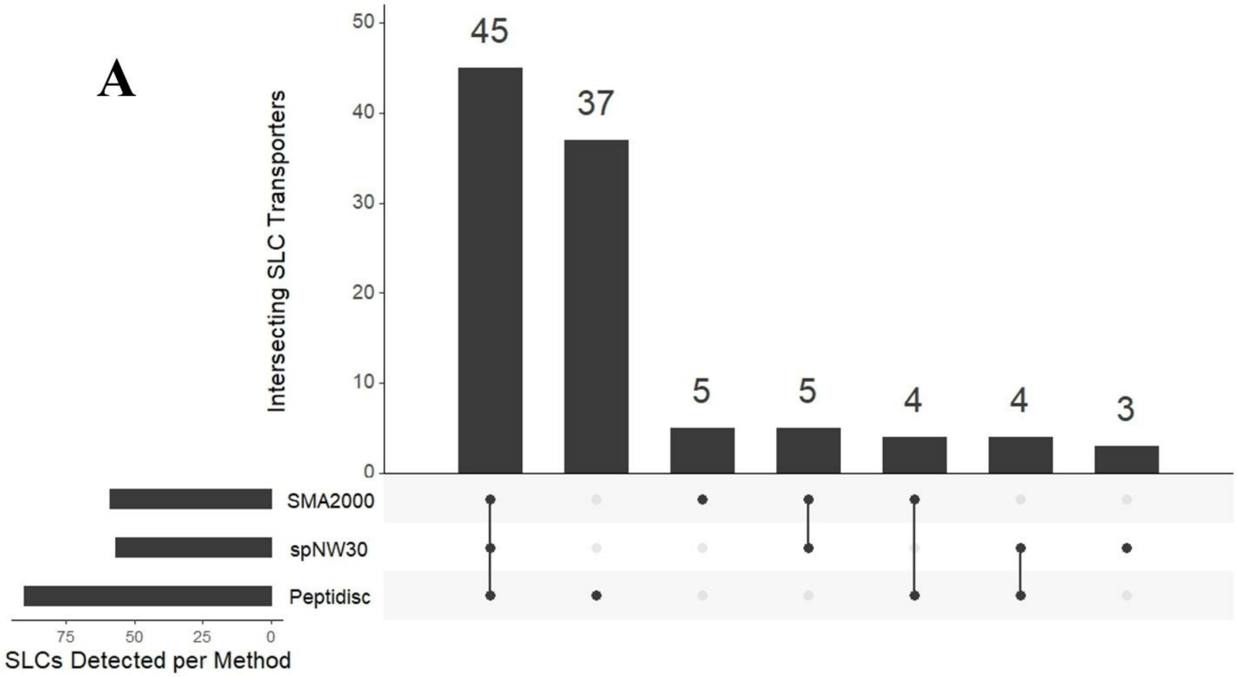
45
37
5
5
4
4
3
Intersecting SLC Transporters
SMA2000
spNW30
Peptidisc
SLCs Detected per Method

B

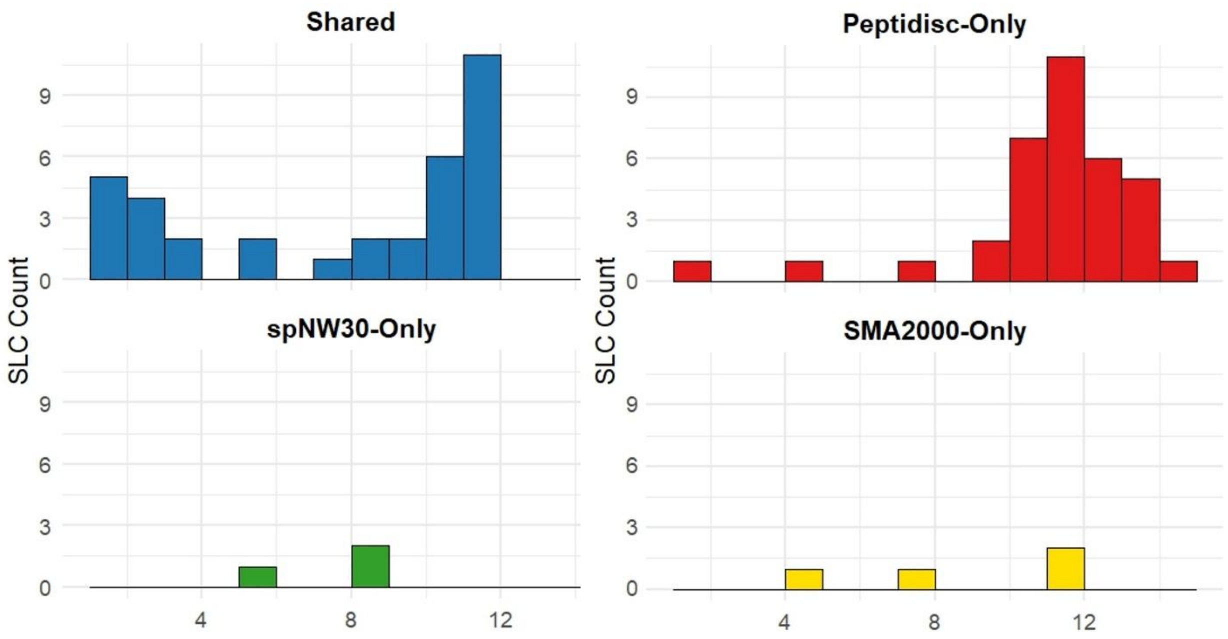
Shared
Peptidisc-Only
spNW30-Only
SMA2000-Only
SLC Count
SLC Count

Supplemental Figure 8. Shared and Unique Detection for SLC Transporters Across Membrane Mimetic Workflow and Transmembrane Segment Distribution

(A) UpSet plot showing the intersection sizes of SLC transporters identified by each method from the replicate with the highest number of protein identifications. SLCs were identified based on the presence of “SLC” in the gene name. Horizontal bars indicate the total number of SLC transporters detected per method. UpSet plots were generated using the ComplexUpset R package.

(B) Histogram showing the distribution of predicted transmembrane segment counts for the SLC shared between Peptidisc, spNW30, and SMA2000 and those uniquely captured by each workflow. Histograms were generated using the ggplot R package.
